# Life’s essential 8 and incident major adverse cardiovascular events in Korea: A nationwide cohort study

**DOI:** 10.1016/j.ajpc.2026.101707

**Published:** 2026-06-18

**Authors:** Sol Hooy Oh, Sang-Suk Choi, Sung-Ho Her, Seung-Hyun Ko, Kyu-Na Lee, Kyung Do Han

**Affiliations:** a Department of Neurosurgery, St. Vincent’s Hospital, College of Medicine, The Catholic University of Korea, Suwon, South Korea; b Division of Cardiology, Department of Internal Medicine, St. Vincent’s Hospital, College of Medicine, The Catholic University of Korea, Suwon, South Korea; c Division of Endocrinology and Metabolism, Department of Internal Medicine, St. Vincent’s Hospital, College of Medicine, The Catholic University of Korea, Suwon, South Korea; d Department of Statistics and Actuarial Science, Soongsil University, Seoul, South Korea

**Keywords:** Life's essential 8, Cardiovascular disease, Major adverse cardiovascular events, Cardiovascular health, Korea, Cohort study

**Abbreviations and acronyms**
**AHA**: American Heart Association
**BMI**: Body Mass Index
**CI**: Confidence Interval
**CKD**: Chronic Kidney Disease
**CVD**: Cardiovascular Disease
**CVH**: Cardiovascular Health
**GPAQ**: Global Physical Activity Questionnaire
**HR**: Hazard Ratio
**ICD-10**: International Classification of Diseases, 10th Revision
**KHEI**: Korean Healthy Eating Index
**KNHANES**: Korea National Health and Nutrition Examination Survey
**LE8**: Life’s Essential 8
**MACE**: Major Adverse Cardiovascular Events
**NHIS**: National Health Insurance Service
**non-HDL**: Non-High-Density Lipoprotein Cholesterol

## 1 Background

Cardiovascular disease (CVD) remains the leading cause of death globally and is rising in Korea with population aging and lifestyle westernization, intensifying the need for primary prevention. In 2022, the American Heart Association introduced Life’s Essential 8 (LE8) [1], an integrated cardiovascular health (CVH) construct comprising four health behaviors (diet, physical activity, smoking, sleep) and four health factors (body mass index, non-HDL cholesterol, blood glucose, blood pressure). Inverse associations between LE8 and cardiovascular outcomes have been demonstrated mainly in Western [2] and Chinese [3] cohorts; however, East Asian populations differ in dietary patterns (higher carbohydrate and sodium intake), a lower prevalence of obesity at equivalent metabolic risk, and population-wide universal healthcare access—factors that may modify the applicability of LE8 and the relative weight of its components.

In Korea, prior work has linked LE8 to all-cause and cardiovascular mortality [4], but its association with incident major adverse cardiovascular events (MACE) has not been examined in a nationally representative cohort with claims-based outcome ascertainment. We therefore investigated the association between LE8 and incident 3-point MACE using nationwide Korean data.

## 2 Methods

In this retrospective cohort study, we linked the Korea National Health and Nutrition Examination Survey (KNHANES) 2014–2018 to National Health Insurance Service (NHIS) claims for longitudinal outcome ascertainment. After excluding individuals under 20 years (n = 7605), pregnant or breastfeeding women (n = 332), participants with missing LE8 components (n = 6655; predominantly the Korean Healthy Eating Index [KHEI, 12.9%], fasting glucose [8.4%], and non-HDL cholesterol [8.1%]), missing covariates (n = 697), prevalent myocardial infarction or stroke (n = 175), and events within a 1-year lag (n = 129), 21,082 participants were analyzed. The study was approved by the Institutional Review Board of St. Vincent’s Hospital (VC24OISI0271); informed consent was waived.

LE8 scores were calculated using AHA algorithms [1] with two culturally adapted instruments: the validated KHEI for dietary quality [5] and the Global Physical Activity Questionnaire. Participants were categorized as low (0–49), moderate (50–79), or high (80–100) CVH per the original AHA framework [1]. Household income (Quartile 1 versus Q2–4) and alcohol consumption (non, mild [<30 g/day men, <20 g/day women], or heavy) were assessed via standardized KNHANES instruments. The primary outcome was 3-point MACE (cardiovascular death, non-fatal myocardial infarction [ICD-10 I21/I22], non-fatal ischemic stroke [I63/I64 with concomitant brain imaging]), ascertained from validated NHIS coding [6]. Multivariable Cox models were adjusted for age, sex, household income, alcohol consumption, and chronic kidney disease; hypertension, diabetes, and dyslipidemia—being LE8 constituents—were not entered as covariates. Component effects were modeled per 10-point increment. Subgroup analyses examined effect modification by age (<65 vs. ≥65 years), sex, household income, and education. Analyses used SAS 9.4 (SAS Institute, Cary, NC, USA).

## 3 Results

Among 21,082 adults (42.2% male; 24.9% aged ≥65 years; mean LE8 score 62.9), participants with higher CVH were more frequently female, college-educated, and in higher income quartiles, with markedly lower prevalence of current smoking, hypertension, and diabetes than the low-CVH group (Panel A).

Over a median follow-up of 4.29 years, 371 MACE events occurred. Incidence per 1000 person-years declined across LE8 categories: low (n = 3769) 7.70, moderate (n = 14,823) 3.81, and high (n = 2490) 0.71. In fully adjusted models, the hazard ratio (HR) was 0.65 (95% CI, 0.52–0.81) for moderate and 0.31 (0.15–0.64) for high versus low CVH (P for trend <0.001); both the health behaviors (HR for high vs. low, 0.50; 0.31–0.81) and health factors (0.64; 0.46–0.90) subdomains were independently protective (Panel B). Kaplan–Meier curves showed graded, dose-dependent cumulative incidence by LE8 category for the composite outcome and for each component—myocardial infarction, ischemic stroke, and cardiovascular mortality (all log-rank P < 0.001; Panel C).

Modeled continuously, each 10-point increment in the Total LE8 score corresponded to a 24% lower composite risk (HR, 0.76; 0.70–0.83; P < 0.001). The signal was strongest for ischemic stroke (0.71; 0.63–0.80), intermediate for myocardial infarction (0.82; 0.70–0.95), and null for cardiovascular mortality (0.91; 0.76–1.09) within this follow-up window. Component-level associations varied substantially: blood glucose (0.92; 0.88–0.96), smoking (0.94; 0.91–0.97), blood pressure (0.95; 0.92–0.98), sleep duration (0.95; 0.92–0.99), and KHEI diet (0.96; 0.93–0.99) were significant for the composite, whereas physical activity, BMI, and non-HDL cholesterol were not—although non-HDL cholesterol was paradoxically associated with higher cardiovascular mortality (1.11; 1.02–1.20) and blood glucose was the strongest predictor of myocardial infarction (0.87; 0.81–0.94) (Panel D).

The Total CVH score was protective across age, sex, income, and education strata (all P-interaction >0.05). By subdomain, the health factors score was significantly modified by age (P-interaction = 0.004) and household income (0.04): strongly protective in adults <65 years (HR for high vs. low, 0.32; 0.18–0.56) and higher-income strata (0.43; 0.26–0.69) but attenuated in older (0.97; 0.63–1.48) and lowest-income (1.01; 0.63–1.64) adults. The health behaviors score remained directionally protective across all strata (Panel E).

## 4 Discussion

In this nationwide Korean cohort, higher LE8 was independently and dose-dependently associated with lower incident MACE, with a 24% relative risk reduction per 10-point increment. This is the first Korean nationally representative analysis linking LE8 to incident MACE ascertained from nationwide insurance claims, extending prior Korean mortality evidence [4] and confirming cross-population concordance with Western [2] and Chinese [3] cohorts and a recent global meta-analysis [7].

The graded effect across outcomes—largest for ischemic stroke, intermediate for myocardial infarction, and null for short-term cardiovascular mortality—suggests that LE8 most strongly captures modifiable atherothrombotic risk and is less informative for the competing-risk-driven cardiovascular deaths observed within a median 4.3-year window. The component hierarchy—glycemic control, smoking, and blood pressure as the strongest predictors—identifies high-yield, actionable prevention targets aligned with the dominant cardiometabolic burden in East Asia, while culturally adapted dietary quality (KHEI) and sleep also contributed independently. The non-significance of BMI and non-HDL cholesterol for the composite, and the paradoxical non-HDL signal for cardiovascular mortality, likely reflect mediation through the already-adjusted glucose and blood-pressure pathways, competing risks, and a follow-up too short to capture lipid- and obesity-mediated atherogenesis; notably, 84.9% of MACE cases were physically inactive versus 71.2% of event-free participants, suggesting a ceiling effect for the physical activity metric in Korea.

From a prevention standpoint, the dominant contributions of glycemic control, smoking, and blood pressure suggest that opportunistic LE8 assessment during routine primary-care visits could help prioritize the modifiable risk factors with the greatest preventive impact in Korean adults. The subgroup attenuation of the health factors subdomain in older and lower-income adults may reflect cumulative legacy exposure and residual social determinants persisting even under universal healthcare, whereas the consistent protection from the overall CVH score and from health behaviors across strata supports lifestyle-centered prevention as broadly equitable.

Limitations include the observational design, single-time-point self-reported behaviors, 18% LE8 missingness driven primarily by complex composite variables rather than systematic differential exclusion, the short median follow-up, and the inability to adjust for other comorbidities (chronic lung disease or liver disease). Nevertheless, our study has several strengths. To our knowledge, it is the first to examine the association between LE8 and incident MACE in a nationally representative Korean cohort, and it further benefits from claims-linked outcome ascertainment, Korean-adapted dietary scoring, and comprehensive component-, outcome-, and subgroup-level analyses.

## 5 Conclusions

In Korean adults, higher LE8 was strongly and independently associated with reduced incident MACE, with glucose control, smoking cessation, and blood pressure management as the priority targets for prevention, supporting the cross-population applicability of the LE8 framework for cardiovascular prevention under universal healthcare Fig. 1.

**Fig. 1:**
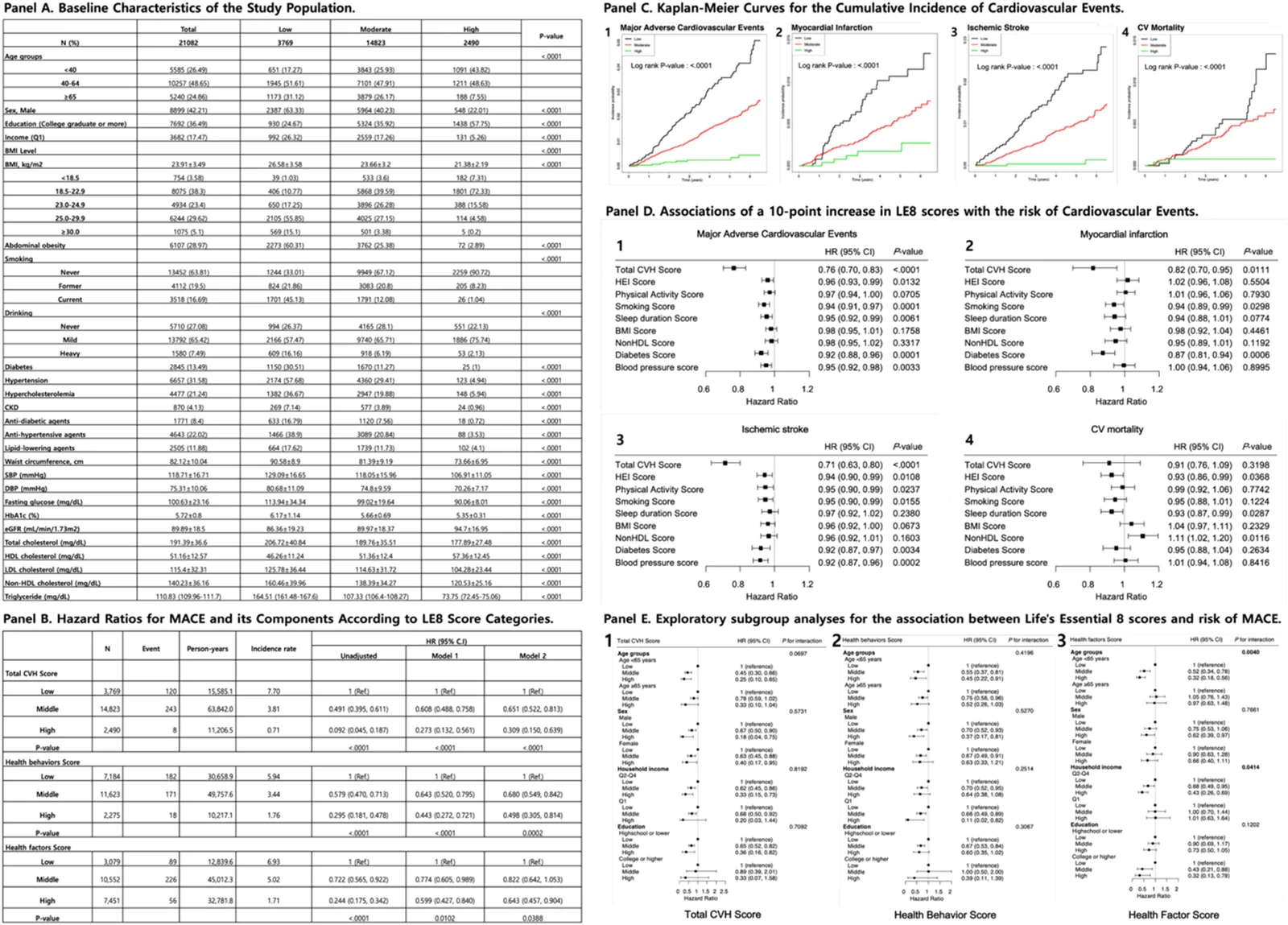
Panel A. Baseline characteristics of the study population stratified by LE8 score category. Values are presented as mean ± standard error for continuous variables and N (%) for categorical variables; group comparisons used analysis of variance or chi-square tests, as appropriate. LE8, Life’s Essential 8; CVH, cardiovascular health; BMI, body mass index; KHEI, Korean Healthy Eating Index; SE, standard error; N, number. **Panel B.** Adjusted hazard ratios for MACE according to Life's Essential 8 score categories (low, moderate, high), shown for the Total CVH score, the health behaviors and health factors subdomains, and each of the eight individual LE8 components, derived from multivariable Cox proportional hazards models with the low category as the reference. The incidence rate per 1000 person-years and the hazard ratio (95% confidence interval) for the moderate and high categories versus low are presented, together with the P-value for trend. HR, hazard ratio; CI, confidence interval; MACE, major adverse cardiovascular events; CVH, cardiovascular health. **Panel C.** Kaplan–Meier cumulative-incidence curves by LE8 category (low, moderate, high), compared using the log-rank test, for (1) the primary composite outcome (MACE; cardiovascular death, non-fatal myocardial infarction, and non-fatal ischemic stroke); (2) myocardial infarction; (3) ischemic stroke; and (4) cardiovascular mortality. The x-axis denotes follow-up time in years and the y-axis the cumulative incidence probability. LE8, Life’s Essential 8; MACE, major adverse cardiovascular events; CV, cardiovascular. **Panel D.** Adjusted hazard ratios per 10-point increment in the Total CVH score and in each of the eight individual LE8 components (KHEI diet, physical activity, smoking, sleep duration, BMI, non-HDL cholesterol, blood glucose, and blood pressure), modeled continuously in fully adjusted Cox models, for (1) MACE; (2) myocardial infarction; (3) ischemic stroke; and (4) cardiovascular mortality. Squares represent point estimates and horizontal lines the 95% confidence intervals; the vertical dashed line indicates a hazard ratio of 1.0. HR, hazard ratio; CI, confidence interval; CVH, cardiovascular health; KHEI, Korean Healthy Eating Index; HEI, Healthy Eating Index; BMI, body mass index; non-HDL, non-high-density lipoprotein cholesterol. **Panel E.** Exploratory subgroup analyses of the association between LE8 scores and MACE, with P-values for interaction, for (1) the Total CVH score; (2) the health behaviors score; and (3) the health factors score. Each subdomain was stratified by age group (<65 vs. ≥65 years), sex, household income (Q2–4 vs. Q1), and education (high school or lower vs. college or higher), with the low category as the reference within each stratum. The health factors score showed significant effect modification by age (P-interaction = 0.004) and household income (P-interaction = 0.04), whereas the Total CVH and health behaviors scores were directionally protective across all strata. HR, hazard ratio; CI, confidence interval; CVH, cardiovascular health.

## Ethics approval

The study protocol was approved by the Institutional Review Board of St. Vincent’s Hospital (VC24OISI0271); informed consent was waived due to the retrospective use of anonymized data.

## Fundings

This research was funded by the Korean Society of Cardiovascular Disease Prevention. The funder had no role in the design, conduct, or reporting of the study.

## Disclosures

The authors declare no conflicts of interest or relationships with industry.

## Data availability

KNHANES data are publicly available (https://knhanes.kdca.go.kr); NHIS claims data are available via formal request to the National Health Insurance Service.

## CRediT authorship contribution statement

**Sol Hooy Oh:** Writing – original draft, Visualization, Methodology, Investigation, Conceptualization. **Sang-Suk Choi:** Writing – review & editing, Supervision, Project administration, Funding acquisition. **Sung-Ho Her:** Writing – review & editing, Validation, Resources, Conceptualization. **Seung-Hyun Ko:** Writing – review & editing, Supervision. **Kyu-Na Lee:** Formal analysis, Data curation. **Kyung Do Han:** Methodology, Formal analysis, Data curation.

## Declaration of competing interest

The authors declare the following financial interests/personal relationships which may be considered as potential competing interests

Sang-Suk Choi reports financial support was provided by Korean Society of Cardiovascular Disease Prevention. If there are other authors, they declare that they have no known competing financial interests or personal relationships that could have appeared to influence the work reported in this paper.
